# Supplementary material for: The interactome of intact mitochondria by cross-linking mass spectrometry provides evidence for coexisting respiratory supercomplexes
Source: Mol Cell Proteomics. 2017 Dec 8;17(2):216–32. doi: 10.1074/mcp.RA117.000470 (PMC5795388; doi:10.1074/mcp.RA117.000470)
Supplement: Supplemental Data [file supp_RA117.000470_133922_0_supp_23978_fzffwf.zip › spectra_annotation/mito_DR_spectra_annotation/55-1-1-1-2-1.pdf]

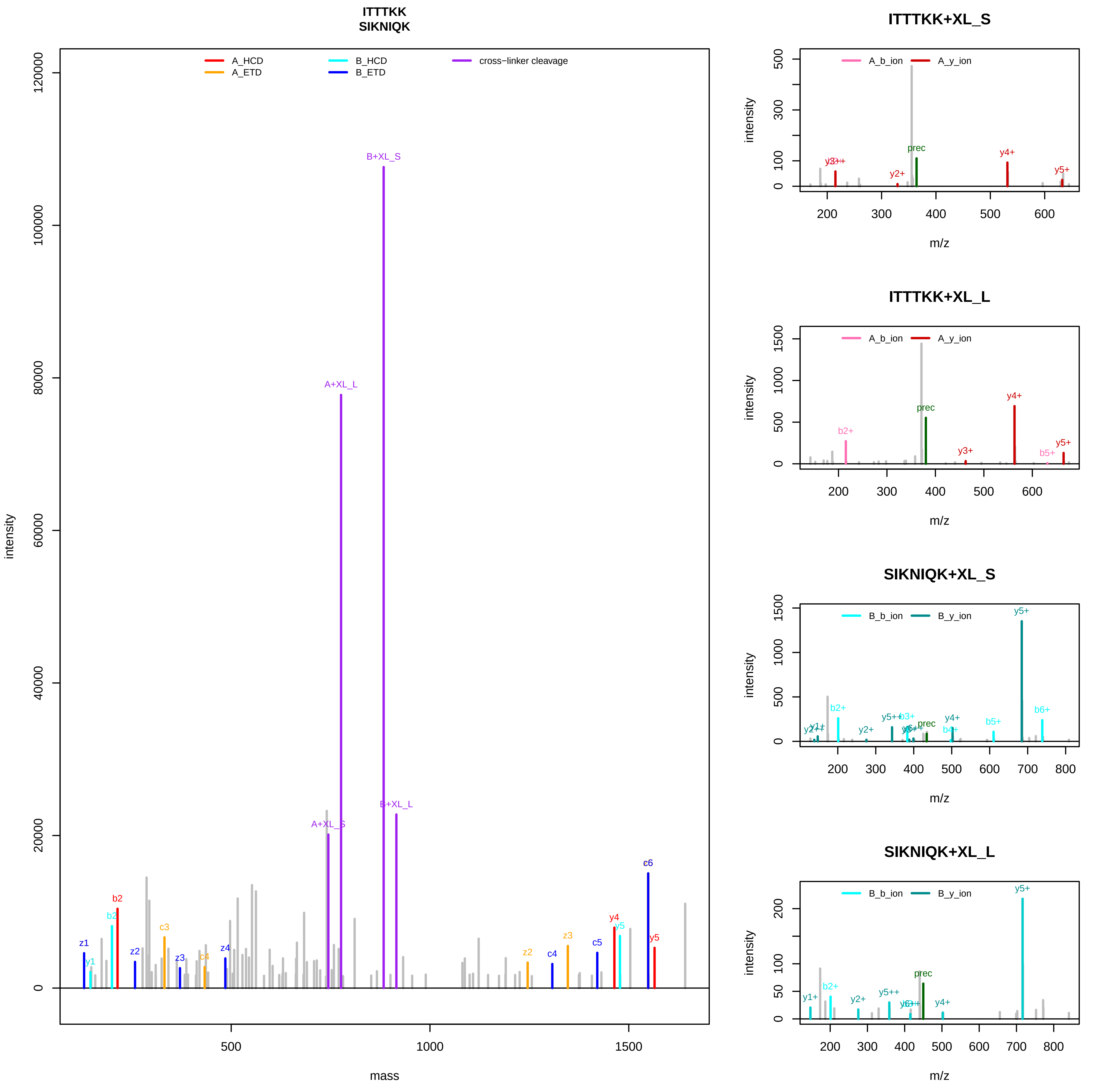

ITTTKK
SIKNIQK
A_HCD
A_ETD
B_HCD
B_ETD
cross−linker cleavage
B+XL_S
A+XL_L
B+XL_L
A+XL_S
z1
y1
b2
b2
z2
c3
z3
c4
z4
z2
c4
z3
c5
y4
y5
c6
y5
intensity
mass
120000
100000
80000
60000
40000
20000
0
500
1000
1500
ITTTKK+XL_S
A_b_ion
A_y_ion
y3++
y2+
prec
y4+
y5+
ITTTKK+XL_L
b2+
prec
y3+
y4+
b5+
y5+
SIKNIQK+XL_S
B_b_ion
B_y_ion
y5+
b2+
y1+
y2++
y2+
y5++
b3+
prec
y4+
b4+
b5+
b6+
SIKNIQK+XL_L
y5+
prec
y1+
b2+
y2+
y5++
y4+
m/z
intensity
